# A Secure Communication in IoT Enabled Underwater and Wireless Sensor Network for Smart Cities

**DOI:** 10.3390/s20154309

**Published:** 2020-08-02

**Authors:** Tariq Ali, Muhammad Irfan, Ahmad Shaf, Abdullah Saeed Alwadie, Ahthasham Sajid, Muhammad Awais, Muhammad Aamir

**Affiliations:** 1 Electrical Engineering Department, College of Engineering, Najran University, Najran 61441, Saudi Arabia; miditta@nu.edu.sa (M.I.); asalwadie@nu.edu.sa (A.S.A.); 2 Department of Computer Science, COMSATS University Islamabad, Sahiwal Campus, Sahiwal 57000, Pakistan; ahmadshaf@cuisahiwal.edu.pk (A.S.); muhammadaamir@cuisahiwal.edu.pk (M.A.); 3 Department of Computer Science, Faculty of ICT, Balochistan University of Information Technology Engineering and Management Sciences, Quetta 87300, Balochistan, Pakistan; ahthasham.sajid@buitms.edu.pk; 4 School of Computing and Communications, Lancaster University, Bailrigg, Lancaster LA1 4YW, UK; m.awais11@lancaster.ac.uk

**Keywords:** topology discover, reliable, routing protocol, smart cities

## Abstract

Nowadays, there is a growing trend in smart cities. Therefore, the Internet of Things (IoT) enabled Underwater and Wireless Sensor Networks (I-UWSN) are mostly used for monitoring and exploring the environment with the help of smart technology, such as smart cities. The acoustic medium is used in underwater communication and radio frequency is mostly used for wireless sensor networks to make communication more reliable. Therefore, some challenging tasks still exist in I-UWSN, i.e., selection of multiple nodes’ reliable paths towards the sink nodes; and efficient topology of the network. In this research, the novel routing protocol, namely Time Based Reliable Link (TBRL), for dynamic topology is proposed to support smart city. TBRL works in three phases. In the first phase, it discovers the topology of each node in network area using a topology discovery algorithm. In the second phase, the reliability of each established link has been determined while using two nodes reliable model for a smart environment. This reliability model reduces the chances of horizontal and higher depth level communication between nodes and selects next reliable forwarders. In the third phase, all paths are examined and the most reliable path is selected to send data packets. TBRL is simulated with the help of a network simulator tool (NS-2 AquaSim). The TBRL is compared with other well known routing protocols, i.e., Depth Based Routing (DBR) and Reliable Energy-efficient Routing Protocol (R-ER$P^{2}$R), to check the performance in terms of end to end delay, packet delivery ratio, and energy consumption of a network. Furthermore, the reliability of TBRL is compared with 2H-ACK and 3H-RM. The simulation results proved that TBRL performs approximately 15% better as compared to DBR and 10% better as compared to R-ER$P^{2}$R in terms of aforementioned performance metrics.

## 1. Introduction

The Internet of Things (IoT) has become the leading technology of growing trends towards smart cities and the environment. To the best of our knowledge, the term IoT is first time coined in 1999 (https://www.postscapes.com/iot-history/ (accessed on 18 June 2020)), which is defined as the infrastructure of the information society. IoUT is defined as the network of smart interconnected underwater objects, which is later known as IoT enabled Underwater and Wireless Sensor Networks (I-UWSNs). The smart objects are the different types of sensors, i.e., Autonomous Underwater Vehicles (AUVs), buoy, ship, watchman nodes, etc. Therefore, I-UWSN architecture is a novel class of IoT and is expected to support various practical applications, such as underwater exploration, environment monitoring, and disaster prevention. With these applications, I-UWSN is considered tp be one of the potential technologies towards the development of smart cities [1,2].

I-UWSN is getting the attention of academia and researchers due to several reasons. One of the main reasons is that the 71% Earth is approximately covered by water and only less than 10% from it is explored. Therefore, it is an emerging technology, which promises the exploration and monitoring of the oceanic environment. There are different ways to deploy sensor nodes in I-UWSN for the aforementioned purposes. The nodes can be randomly deployed or in the form of a grid and tree-structure. Firstly, to provide reliable communication, each sensor node has computational, communication, and intelligence capabilities to deal with a smart environment, as in smart cities [3,4]. Secondly, node sense the data and flood it towards the destination node. State of the art I-UWSN applications are environmental monitoring, disaster warning, assisted navigation, natural resources exploration, and military applications [4,5,6].

Several routing protocols have been proposed for the aforementioned applications. Existing terrestrial network routing protocols cannot be used in underwater and smart city communication [7,8] due to their unique characteristics of communication medium, e.g., the limited bandwidth due to the uses of the acoustic medium. The speed of acoustic medium is five magnitudes less when compared to that of the radio frequency; therefore, the magnitude of propagation delay occurs in underwater environment is five times higher when compared to terrestrial environment. High bit error rates and momentary losses of connectivity (shadow zones) could occur and the power of the the battery suffers shortage, especially batteries cannot be recharged frequently [9,10,11]. Different I-UWSN routing protocols are introduced to overcome these challenges by taking the advantages of underwater characteristics. Each schema has both achievements and deficiencies. Therefore, the design and implementation of routing protocol is an active research area of I-UWSN to favor the smart city idea.

Thirdly, the routing protocol must ensure the reliability and effectiveness of data transmission from the source to the destination node. On behalf of reliability, the application goals can be reached or lead to the right directions or decisions. Therefore, reliable communication that is required for every application either exists in WSN or I-UWSN [12]. In both of the networks, it is a challenging task to make an application reliable. In I-UWSN, sensor nodes move from one to four meters per second because of water currents, i.e., water pressure, temperature, salinity, and water bodies movement. Because of these movements, the dynamic topology needs to overcome the causes that decrease the reliability of routing [6,13]. In [14], two approaches are introduced that enhance the reliability of data Forward Error Correction (FEC) and re-transmission-based schema. In FEC, redundancy is added for transmission of data packets; whereas, for the re-transmission of data, sender request is served by Automatic Repeat Request (ARQ) and retransmitted the failed packet.

Reliable link obtains network topology, which, in turn, determines the energy consumption, throughput and transmission delay of the network [15]. The stable connection consist of packet based (all generated packets are reliable), the second is event based (all created events for routing process is reliable), and the third is the summation of packet+ event (packets generated from a source node by maintaining a reliable link towards destination node) [14]. A reliable link must be considered as the selection of the path that has a higher probability of success ratio [16,17]. As environmental monitoring systems and applications are becoming popular with time and are now in high demand for smart cities. In this paper, we have proposed state of the art protocol Time Based Reliable Link (TBRL), in which the distance-based reliability mechanism is used to reduce the effect of dynamic topology. The TBRL measures link quality for all existing routing paths from source to destination and selects one of the best paths for data transmission. By taking the benefits of limited resources, a flexible and robust mechanism for searching a path that is more reliable in terms of data, energy, delay, and throughput is considered in TBRL. The following are expected outcomes of the proposed TBRL:

- Positioned aware routing without using Global Positioning System (GPS).
- Node to node reliability assurance.
- All possible paths are extracted from offshore to sink and less. effected path is taken for sending the data.
- Expected delay and energy consumption of network are well known before the simulation s are conducted.
- No complex routing table is maintained and no additional hardware resources are utilized for TBRL.
- TBRL is applicable for smart environment like smart cities to provide a reliable path for communication between sensors and smart devices.
- TBRL provides help to the machine learning approach for underwater object identification and classification.

We have organized this paper, as follows: in the Section 2, related work is given; system model is presented in the Section 3 and Section 4 explains the mathematical model; in the Section 5, simulation results are described. Finally, we concluded the paper in Section 6.

## 2. Related Work

Many routing protocols have been proposed in the last decade to provide reliable communication in an underwater and smart environments. As in [2], to address the issues of network recovering delay and design complexity a Self-Organized Underwater wireless sensor Network (SOUNET) is proposed. It uses tree topology for the deployment of nodes. Dynamic addresses are assigned to each node for their identification, hop count, or hierarchical level. For the addition of a new node at any level, only a parent address is required. It also solves the nodes isolation problem and closed loop, but SOUNET is not capable enough to provide reliable communication for a long routing path. In [3], a detailed description of localization based and localization free UWSNs routing protocols are discussed. Protocols that require prior network information before starting routing is called localization based, whereas localization free methodology is opposite to it. Every designed routing protocol for underwater communication has its limitations in terms of reliability, delay, data packets losses, and throughput issues. In [5], a comprehensive survey of existing UWSNs applications, their deployment challenges and opportunities in UWSNs environment are presented by the authors. They have gathered the data of existing applications and divided it into five major fields that are furthered divided into relevant subclasses.

### Reliable and Topology Control Protocols in Uwsns

In [4], the Topology Efficient Discovery (TED) routing protocol is introduced. TED not only reduces time overhead, but also accurately evaluates link reliability. TED is based on time windows allocated for transmission in which a time slot is defined for nodes to transmit a packet or several packets. By using a novel algorithm Near-Far Node Pairs (NFNPs), it also supports for discovering node pairs whose transmission is blocked. In [8], analytical work to measure the link reliability is shown. Based on their model, any UWSNs system‘s reliability can be analyzed. Their model supports Binary Phase Shift Key (BPSK), Multiple Input Multiple Output (MIMO), and Code Division Multiple Access (CDMA) communications system. In [14], erasure coding of end-to-end and hop-by-hop are evaluated for improving the data reliability of multi-hop UWSN. Depth Based Routing (DBR) protocol is used for the routing process. DBR has an efficient approach to handle the network dynamically without any localization information.

In [16], traversal and radii incremental are two algorithms that are proposed for topology control in diverse coverage. In the traversal approach, at each round, only sensing node increases its radii, whereas in the radii incremental approach, multiple nodes radii increase simultaneously at each round. In [17], Channel Aware Routing Protocol (CARP) is proposed for exploiting link quality information. Only those nodes are selected for data forwarding that has successful transmission history to their neighbors. In this way link quality information exploits. It uses hop count for managing topology of the network. In [18], an energy management policy is introduced by the authors for maximizing the network performance and network lifetime. They used a genetic algorithm based meta-heuristic search for global design space and local pattern search algorithm for finding an optimal solution.

In [19], a sphere energy depletion model and nodes mobility are taken into consideration for balancing the energy consumption of nodes in I-UWSN. Routing voids and redundancy of data packets are also controlled by their schema. Further, they have explained the tradeoff between data delivery reliability and energy efficiency. In [20], high bit error rate and a void problem cause the acoustic transmission to be unreliable in an underwater environment. To overcome this issue, intermediate nodes collaboration for forwarding the data packets is used. The best forwarding path is selected by applying a low-cost heuristic search. In [21], a reliable and energy efficient protocol is proposed by authors that is based on physical distance, link quality, and residual energy information. A significant amount of improvement is considered as compared to their previous work with respect to end-to-end delay, network lifetime, energy consumption, and delivery ratio. Furthermore, the comparison of some state of the art routing protocols is given in Table 1.

## 3. System Model

**Figure 1:**
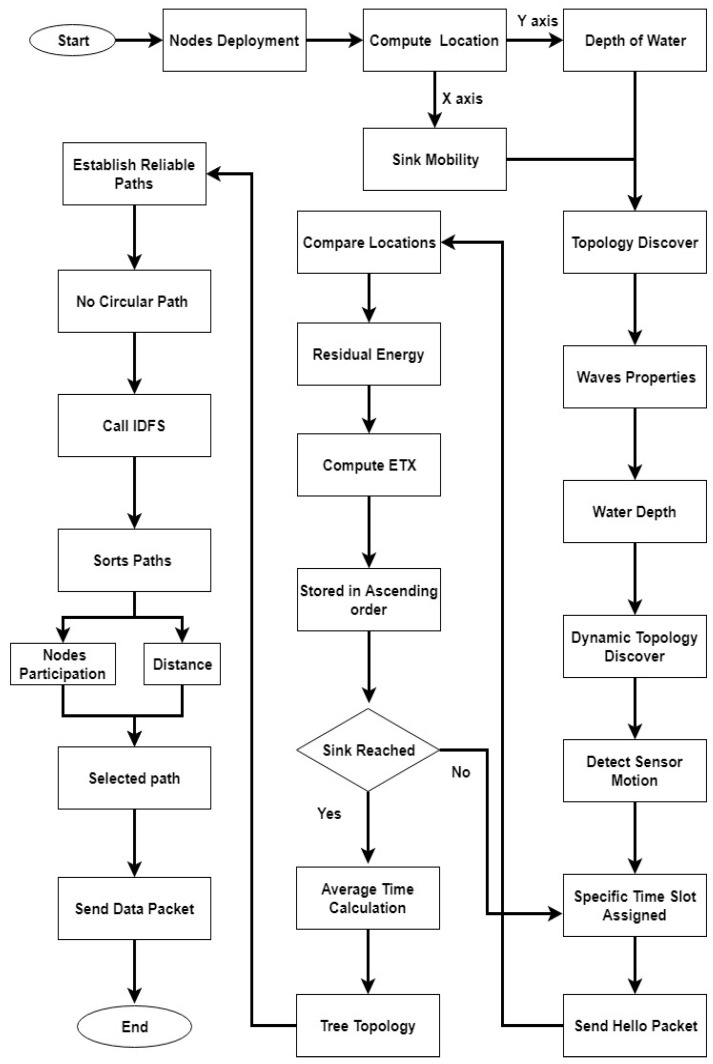
Flow Chart of Time Based Reliable Link (TBRL).

A novel routing protocol, called TBRL, for dynamic topology in critical underwater monitoring missions is proposed. In TBRL, reliable links are established between the nodes at different depth levels. It is a reliable, delay sensitive, energy efficient, and efficient topology discovery routing protocol that uses multi-sink architecture. Several surface sinks are used to collect data that are being sent from sensor nodes on the water surface and some nodes are anchored at the bottom. The remaining nodes are deployed at different levels between the water surface and the ground. The nodes will forward data packets that are based on a link quality. By considering reliability, any node used for the data collection delivers the data towards the next nodes. Data packets that reach any of the surface sinks are considered to be successfully delivered to the final destination. Figure 1 shows the flow chart of TBRL. These sinks have the facility of radio communication, which enables them to communicate with each other at higher bandwidths and lower propagation delays.

TBRL completes its communication process in three phases:

- dynamic topology discovery algorithm,
- two nodes reliable model, and
- topology changes of existing paths.

Phase one is to assign nodes’ location by considering their depth and water speed at different depth levels. In the second phase, nodes forward the data packets towards the next nodes or sinks by ensuring the reliability of a link. In the third phase, all of the possible reliable paths are taken as an input parameter and return the less affected path.

### 3.1. Dynamic Topology Discovery Algorithm

**Figure 2:**
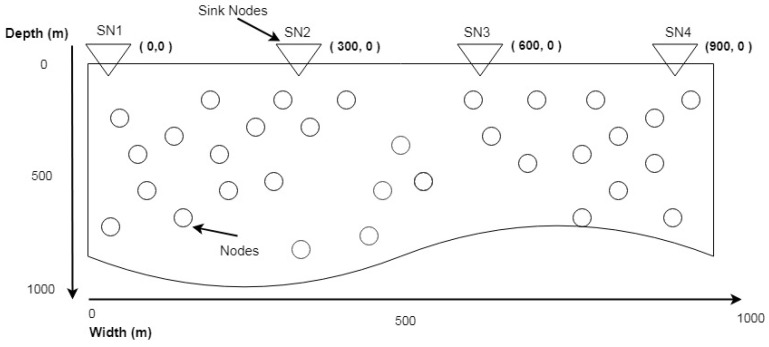
Finding X axis Location.

TBRL is a positioned aware routing protocol. To obtain nodes’ position; initially, we assume that the X axis of each node is unknown and Y axis is equal to the depth of water. Due to the continuous flow of water, the X axis of each node changes its position frequently when compared to its Y axis. Further, the oceanic environment depth (Y axis) calculation is far easier as compared to the width (X axis) calculation. Multiple sinks that are placed at equal distance and random deployment of nodes are shown in Figure 2. Furthermore, special mobility as per the area of network has been assigned to each sink from its initial point to the last point and that last point is the initial point of the next sink. Sink nodes move from their initial point to another point by calculating their movement towards X axis. Because of the horizontal movement of sink nodes, the Y axis is always zero and it cannot be updated. Therefore, distance always changes in X direction.

**Figure 3:**
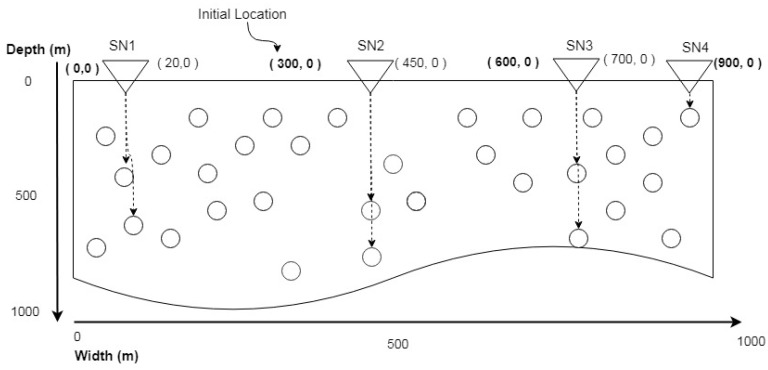
Assigning X axis Location.

X axis is stored in a Location Packet (LP) and sends the constant value of X axis vertically. In this way, nodes become well aware of their X axis location in the network. Nodes determining location process are also depicted in Figure 3. Sink Node 1 (SN1) has an initial location (0,0), it will send LP downward by taking the X axis 0 and increasing the value of Y axis. If a node receives LP, it will update its X axis location that was unknown to it at the time of deployment. Similarly, SN1 sends LP at different X axis and updates the location of downward node. SN1 can move from 0 to 300 m, which goes back to its initial point after reaching a final point.

Other approaches to find the horizontal distance are not suited in this environment. At same depth level, the distance would be smaller or larger; therefore, Pythagoras theorem and angle based side calculation mechanism did not perform well in such a harsh environment. Algorithm 1 is proposed instead of GPS. Because of the moment of water, nodes move from 1 to 4 m/s and that movement varies from depth to depth. Wave theory helps us to understand such movement at a specific depth level. Waves are described in terms of length, height, and period. Because water waves move in a circular shape and its crest is approximately 120 degrees. With the increasing depth of water, its wave height becomes shorter and wavelength becomes longer. Consequently, higher pressure and speed exist in deep water. Fetter and Walecka proposed Equation (1) to calculate the speed of water.

(1) $$speed=\sqrt{(}\left(\left(g*\lambda \right)/\left(2*\pi \right)\right)*tanh\left(2*\pi *h/\lambda \right))$$

Here, *g* is the gravitational force, λ is the wavelength and *h* represents depth of water. For λ, we used Equation (2).

(2) $$\lambda =\frac{1}{2}*h$$

We observed this equation in the small tub. However, this equation fails to determine the speed in large area. To do this, the properties of waves are taken into consideration. By using them, the wavelength of waves at a large scale is determined.

(3) $$\lambda =\frac{HC}{2h}$$

*H* presents wave height and *C* shows speed of wave. To compute the value of *H*, Equation (4) is used.

(4) $$H=\frac{\frac{1}{2}*h}{\sqrt[{4}]{\frac{8*\pi *h}{L}}}$$

*L* and *h* represent wave length and water depth to determine the value of *L*.

(5) $$L=L_{o}\sqrt{tanh(2*\pi \frac{h}{L_{o}})}$$

$L_{o}$ is the speed of wave at H.

(6) $$L_{o}=\frac{C^{2}*2*\pi }{g}$$

(7) $$C=\sqrt{gh}$$

(8) $$L_{o}=\frac{{\sqrt{gh}}^{2}*2*\pi }{g}$$

(9) $$L_{o}=\frac{gh*2*\pi }{g}$$

(10) $$L_{o}=2*\pi *h$$

After putting values of $L_{o}$ into Equation (5).

(11) $$L=2*\pi *h\sqrt{tanh(2*\pi \frac{h}{2*\pi *h})}$$

(12) $$L=2*\pi *h\sqrt{tanh(1)}$$

(13) $$L=2*\pi *h\sqrt{0.76}$$

(14) L=2∗π∗h∗0.87

(15) L=1.74∗π∗h

Putting the value of *L* into Equation (4).

(16) $$H=\frac{\frac{1}{2}*h}{\sqrt[{4}]{\frac{8*\pi *h}{1.74*\pi *h}}}$$

(17) $$H=\frac{\frac{1}{2}*h}{\sqrt[{4}]{4.5}}$$

(18) $$H=\frac{\frac{1}{2}*h}{1.45}$$

After simplifying, *H* becomes

(19) H=0.34∗h

Put the vale of *H* and *C* into Equation (3), λ is calculated as:

(20) $$\lambda =\frac{0.34*h*\sqrt{gh}}{2h}$$

(21) $$\lambda =0.17*\sqrt{gh}$$

After the deployment of nodes, sink nodes can move from their predefined to next position with the help of Algorithm 1. With the help of sink nodes, each node discovers its approximate X axis location, whereas the Y axis of each node is measured through the depth of water in an underwater environment. Here, *g* is the gravitational force, p_time is the previous time when topology is changed, *x* and *y* present the x and y axis of node, λ shows the wave length, speed presents the water speed at current depth level, time determines the time of last modification with respect to the current time, and this helps us to calculate the current location of a node. Because waves move in a circular shape and circle covers $0^{\circ }$ to $360^{\circ }$. Each circle’s center point is the origin of quadrant and it covers all four quadrants; therefore, speed is divided by 4. This algorithm helps us to find the approximate change of node movement either in forward or backward direction. After calculating waves speed at current depth level and time elapsed, we find a certain node movement while using step 11 of Algorithm 1.

### 3.2. Two Nodes Reliable Model (2n-Rm)

For establishing reliable links, 2N-RM is introduced in which three important factors are considered distance, Expected Transmission Count (ETX), and residual energy.

#### 3.2.1. Distance

Each sensor node has certain range limit that is assigned by routing protocols. By default, in AODV, DSDV, and DSR protocols, sensor nodes’ range limit is 250 m; whereas, in the VBF and DBR protocols, nodes range limit is 100 m. Consequently, destination or intermediate node must reside in this range to take data from the source node. In TBRL, nodes’ range limit is set to 70 m and computes Euclidean distance between the source to the next forwarder.

(22) $$\begin{array}{c} Distance=\sqrt{{\left(X_{2}-X_{1}\right)}^{2}+{\left(Y_{2}-Y_{1}\right)}^{2}},\\ 70>Distance>0 \end{array}$$

$X_{2},X_{1},Y_{2}$, and $Y_{1}$ present the position of node2 X, node1 X, node2 Y, and node1 Y, respectively. The reliability of the links is inversely proportional to the distance between the nodes. Less distance means higher reliability while higher distance means less reliability. ge limit is set to 70 m and computes Euclidean distance between source to the distance must be greater than 0 and less than 70 m to maintain ge limit is set to 70 m and computes Euclidean distance between source to a reliable link. The reliable link must fulfill the given condition. The ge limit is set to 70 m and computes Euclidean distance between source to the successful link usually means finding a resource with excellent exactitude and recall, and retrieving an authentic representation of the resource in a timely fashion, i.e., limit is set to 70 m and computes Euclidean distance with sufficiently low latency, reduced bit error rate. The parent is chosen between neighbors consistent with link estimation ways and distance to place the reliable links in routing tables instead of those supported hop count. The used metrics explore cross-layer information to boost dependableness, and also the links between neighbors are evaluated in an exceedingly probabilistic and accommodative manner to gauge even not already chosen nodes with short searching and passive link observation ways.

**Algorithm 1** Dynamic Topology Discovery
**Input:** All nodes from source to sinks
**Output:** Determine each node location
    *Initialization*:
1:g=9.81         // Gravity remains same in air and water2:p_time=0         // All nodes previous time3:**for** i=1 to all nodes **do**4:    x=LP[i]         // Location Packet5:    y=Depth_of_node[i]6:    $\lambda =0.17*\sqrt{gy}$
7:    $speed=\sqrt{(}\left(\left(g*\lambda \right)/\left(2*\pi \right)\right)*tanh\left(2*\pi *y/\lambda \right))$8:    x1=speed/4
9:    time=current_time−p_time
10:    x2=time∗x111:    node[i] ((x±x2),y)12:    p_time=current_time
13:**end for**


#### 3.2.2. Expected Transmission Count

Another important factor to measure link reliability is ETX. It is a probability of successful packet delivery ratio and its acknowledgment over a link. ETX is computed through equation that is given below:

(23) $$ETX=\frac{1}{Sr*Ar}$$

Sr and Ar represent the packet sending ratio and packet acknowledgment ratioge limit is set to 70 m and computes Euclidean distance between source to, respectively. ETX depends upon the delivery ratio, which directly relates to the throughput of network. It also determines the path loss ratio and selects the optimal route for the packet.

#### 3.2.3. Residual Energy

One more factor is to calculate the energy status of the sensor node. All of the participating nodes must be alive and have a sufficient amount of energy for sending and receiving the data packets. A reliable link from source to sink must consist of all alive nodes.

#### 3.2.4. Reliable Paths Establishing

**Figure 4:**
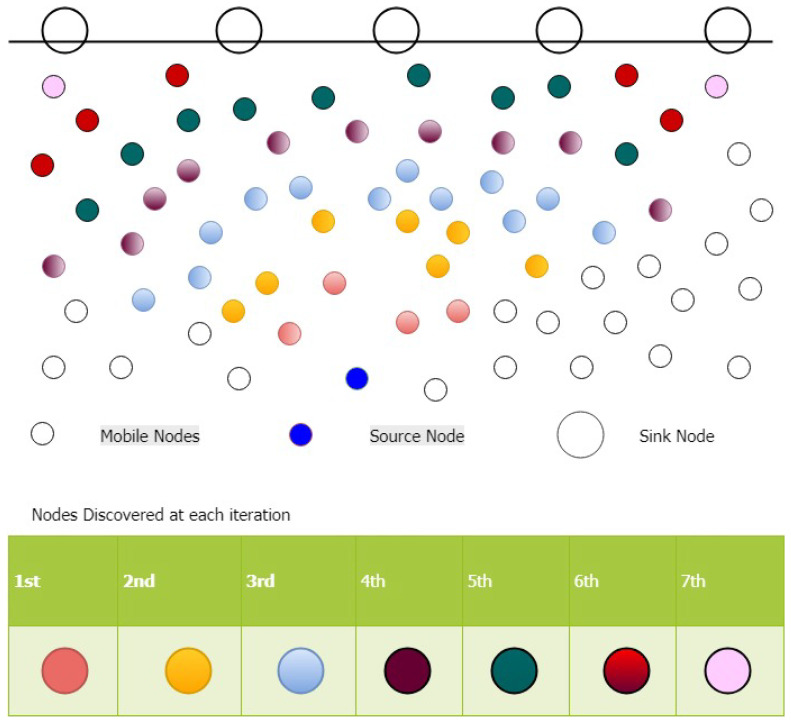
Reliable Nodes Discovery.

By using 2N-RM, all of the selected nodes become source nodes and start discovering other reliable nodes. Consequently, multiple reliable receivers are being found and it continues until the sink nodes reached. Reliable nodes discovery from a single source to multiple sinks are depicted in Figure 4.

**Figure 5:**
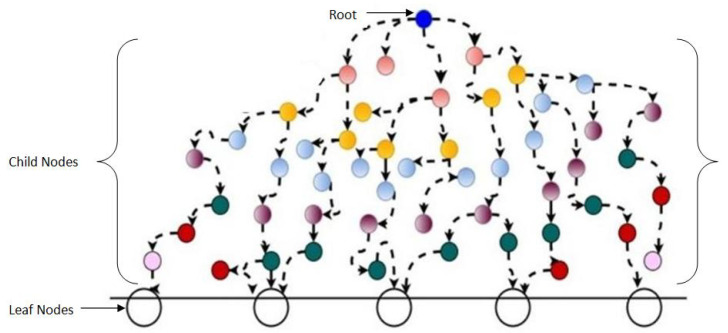
Tree Topology of Establishing Paths.

After discovering the reliable nodes, multiple paths are established from source to sink nodes. We converted our reliable nodes into tree topology. In this topology, the initial source node becomes a root node and all sinks behave as a leaf node. Multiple intermediate nodes to make a path from root to leaf nodes are considered as child nodes. Tree topology of reliable paths is shown in Figure 5. At each step, it can be seen that each child has only one parent, there is neither any loop, nor any circuit. In case of multiple request receiving, a node selects parent on the basis of first come first serve. Due to acoustic transmission, nodes that are closer to each other have efficient energy consumption, delay, throughput, and reliable data transmission.

#### 3.2.5. Selection Process of Reliable Link

In 2N-RM, source node sends hello packet towards its neighbors. Hello packet format only consists of node id. Nodes receive hello packet and send the acknowledgment to source node. Acknowledgment packet format consists of receiver node id, location, and residual energy. After that, source node calculates physical distance and compares its location with other nodes. Nodes having higher Y axis and their distance is greater than 70 m will be discarded from the queue. Link has been established from source to the selected nodes and reliability of each node is measured through ETX value by sending 10 hello messages after a specific time interval. Sr and Ar are calculated and the resulted value of each link is stored in a queue. All of the results are saved in ascending order. Link has closer value to 1 will be selected as a most reliable route.

**Figure 6:**
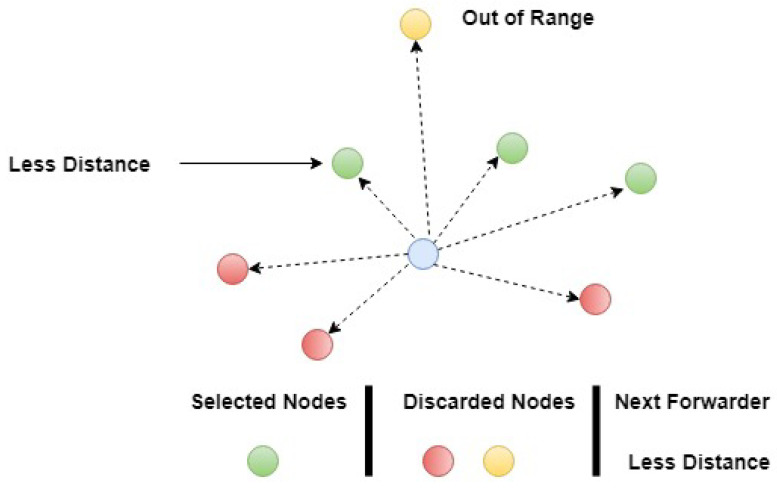
Next Forwarder Selection.

In the case of an infected node or a node having lower residual energy, such type of sorting reduces the time that is required by 2N-RM for the selection of other nodes. By taking the advantages of tree properties, we find the optimal path with respect to the maximum number of nodes participation. For this purpose, the Iterative Depth First Search (IDFS) algorithm is used. IDFS sorts the paths and provides us the optimal path. After selecting the optimal path, data packets will be forwarded towards the sink node. Nodes residing in predefined limit have approximately same ETX value and, due to harsh underwater environment, a node having lower distance as compared to other will be selected (see Figure 6).

### 3.3. Dynamic Topology Discovery of Existing Paths

In this section, Algorithm 2 is proposed for TBRL to check the affected paths through dynamic topology. Initially, all of the paths from source to different sinks are taken as an input parameter. All of these paths had been created by different nodes. At step 2, each path’s nodes has been extracted and saved in an array. Multiple nodes are placed inside the array, after that, each node’s speed has been calculated. At speed determining step, *g* is a gravitational force that is caused by water, “lambda” is the wavelength of wave determined in Equation (21), and *h* is the depth of the node. TBRL is a positioned based routing technique; therefore, each node knows its location and depth level. At step 6, the node’s new position is saved in another array. After the completion of outer loop, all new paths have been taken out and passed in “Paths”. These paths are further assigned to the source node, and then IDFS will be called and it will select the path that have maximum number of intermediate nodes from source to sink node.

**Algorithm 2** Dynamic Topology Discovery of Existing Paths
**Input:** All Paths from source to sink nodes extracted through IDFS
**Output:** Effected Paths due to topology
    *Initialization*:
1:**for** i=1 toAllPaths **do**
2:  Path_Nodes [ ] = i        // Nodes that make a path3:  **for** j=0toPath _ Nodes **do**4:    $speed=\sqrt{(}\left(\left(g*\lambda \right)/\left(2*\pi \right)\right)*tanh\left(2*\pi *h_{j}/\lambda \right))$5:    move=speed/4
6:    $node_{ii}=$$node_{j}$((X±move),Y)       // Topology Changed7:    $node_{j}=node_{ii}$
8:    new _ Path _ $Node\;\left[\;\right]=node_{j}$9:  **end for**10:  $Path_{i}=new$ _ Path _ Node[]11:**end for**12:**return**$Paths_{i}$// Paths received from step 12 will be entertained in given steps13:**for** m=1toPaths **do**
14:  Call IDFS(m)15:  Compare Number of Nodes Participating16:  Select path having higher nodes participation17:**end for**


## 4. Mathematical Model

Two mathematical models for assigning time slot and energy consumption are discussed below. This section explains the time slot that is required by a source node for forwarding the data packets to sink node. At first iteration, initially, $t_{0}$ *s* is taken as a specific time slot. Source node sends hello packet and waits for $t_{0}$ *s* for taking acknowledgment from other nodes. Because multiple receivers reply in that time; therefore, average time is calculated. This average time is used for the time slot allocation of next iteration.

(24) $$t_{1}=\frac{\sum NRT}{NN}$$

where, ∑ *NRT* represents the summation of all nodes’ reply time and *NN* is number of nodes that are replied. Similarly in the next iteration, multiple nodes become source nodes and create a reliable link. In Equations (25)–(28), there are two summation symbols used: the first symbol is used to add all receivers‘ reply time while in the second symbol limit is assigned from 1 to *n*, where *n* represents the total number of source nodes found in the previous iteration and outer summation sign acting as a loop.

(25) $$t_{2}=\sum_{i=1}^{n}\frac{\sum NRT_{i}}{NN_{i}}$$

Average time required by source nodes for *n* iterations is calculated as:

(26) $$t_{n}=\sum_{i=1}^{n}\frac{\sum NRT_{i}}{NN_{i}}$$

Summation of average time for *n* iterations is written as:

(27) $$t_{t}=t_{1}+t_{2}+\ldots t_{n}$$

All corresponding values are placed inside the Equation (28)

(28) $$\begin{array}{cc} t_{t} & =\frac{\sum NRT}{NN}+\sum_{i=1}^{n}\frac{\sum NRT_{i}}{NN_{i}}+\ldots \sum_{i=1}^{n}\frac{\sum NRT_{i}}{NN_{i}} \end{array}$$

*m* represents the number of nodes in network.

(29) $$t_{t}=\frac{\sum NRT}{NN}+\sum_{k=1}^{m}\left[\sum_{i=1}^{n}\frac{\sum NRT_{i}}{NN_{i}}\right]$$

The results of $t_{1}$ can also be calculated through next term, which is the summation of average node reply time. Hence, the average time that is required by a source node to reach at any sink node is calculated through Equation (30).

(30) $$t_{t}=\sum_{k=1}^{m}\left[\sum_{i=1}^{n}\frac{\sum NRT_{i}}{NN_{i}}\right]$$

### 4.1. Energy Consumption

To calculate the energy consumption of our model, we have considered the parameters that are discussed in Table 2.

#### 4.1.1. Energy Consumed by Hps, Hpr and Hpra

HPR is taken twice as compared to HPS and HPRA due to the energy consumption of HPR at both ends when receiving. Energy that is consumed at each iteration in the form of HPS and HPR is written as:

(31) $$E_{1}=HPS+2HPR+HPRA$$

For *N* number of nodes, energy consumption is calculated as:

(32) $$E_{N}=N\left(HPS+2HPR+HPRA\right)$$

#### 4.1.2. Energy Consumed by Dpe

Data packet is forwarded to selected node which is reliable in terms of distance; therefore, energy consumed by *DPE* is calculated as:

(33) $$E_{1}=DPE$$

For *TD* and *N*, energy consumption is calculated as:

(34) $$E_{N}=N\left(TD*DPE\right)$$

Combining Equations (32) and (34).

(35) TE=N(TD∗DPE)+N(HPS+2HPR+HPRA)

*HPS* and *HPRA* energy consumption remain same and *HPR* is taken one over fifth of *HPS*. Now, Equation (35) is written as:

(36) $$TE=N\left(TD*DPE\right)+N\left(HPS+\frac{2HPS}{5}+HPS\right)$$

(37) TE=N(TD∗DPE)+N(2.4∗HPS)

(38) TE=N(TD∗DPE)+N(2.4∗HPS)

(39) TE=N((TD∗DPE)+2.4∗HPS)

Energy is also affected by ship turbulence, water current movement, wind speed, biological noises, etc. Therefore, Signal to Noise Ratio (SNR) is also added into total energy. *SNR* is calculated by the following equation.

(40) TE=N((TD∗DPE)+2.4∗HPS)+SNR

(41) SNR=SL−TL−NL+DI

where, *SL* is the target noise level of source node, *TL* is transmission loss, *NL* is noise level from receiver plus environment, and *DI* is directive index. Values of *NL*, *DI*, and *SNR* are taken from [34]. Their values for deep water are given in Table 3. *TL* for deep sea is given by the following equation.

(42) $$TL=20logd+\alpha *d*10^{-3}$$

*TL* is directly proportional to distance dependent attenuation *d* and frequency dependent absorption α. Thorp’s expression calculates α for frequencies above few hundred Hertz through following equation.

(43) $$\alpha =\frac{0.1*f^{2}}{{\left(1+f\right)}^{2}}+\frac{40*f^{2}}{4100+f^{2}}+2.75*10^{-4}f^{2}+0.003$$

For lower frequencies. α is given by equation.

(44) $$\alpha =\frac{0.11*f^{2}}{{\left(1+f\right)}^{2}}+0.011f^{2}+0.002$$

where, α is measured in dB/km and *f* is in kHz. SL is directly related to signal intensity at 1 m from where the signal is generated and can be calculated, as follows.

(45) $$SL=10log\frac{I_{T}}{1\upsilon P_{a}}$$

For deep sea source, transmission power $P_{T}\left(d\right)$, which is in $Watts/m^{2}$, is calculated as:

(46) $$P_{T}\left(d\right)=4\pi *{\left(1m\right)}^{2}*I_{T}$$

By Equation (40), the total energy consumption of a network having *N* number of nodes at different depth and *TD* are calculated.

## 5. Simulation and Results

In this section, we evaluated the performance of TBRL routing protocol through simulations. To highlight the outstanding qualities of the TBRL, we combined the 2N-RM with the TBRL and compared the performance of TBRL with DBR and Reliable Energy-efficient Routing Protocol (R-ER$P^{2}$R). The simulations’ setting and results are discussed in the given section.

### 5.1. Simulation Setting

NS-2 with Aquasim package is used for the evaluation of TBRL. Taking the area of 1000 × 1000 m${}^{2}$, 500 mobile nodes were randomly deployed inside the water and multiple sink nodes are placed at the water surface. The sink nodes are equipped with radio and acoustic type of communication medium. Sink nodes considered to be static throughout the simulation environment, but remaining nodes can move from 1 to 4 m/s inside the water. The diagonal and vertical movement of nodes is ignored. Only horizontal movement of nodes is considered for simulation environment. Different set of nodes are tested against TBRL. Two nodes placed at the seabed and selected as source nodes. The data packet size is fixed at 1000 bytes. Each source node generates a constant bit rate traffic. The simulation results are taken from the average value of 50 runs for each node dataset. Table 4 also shows the simulation setting provided to TBRL.

### 5.2. Results and Discussion

**Figure 7:**
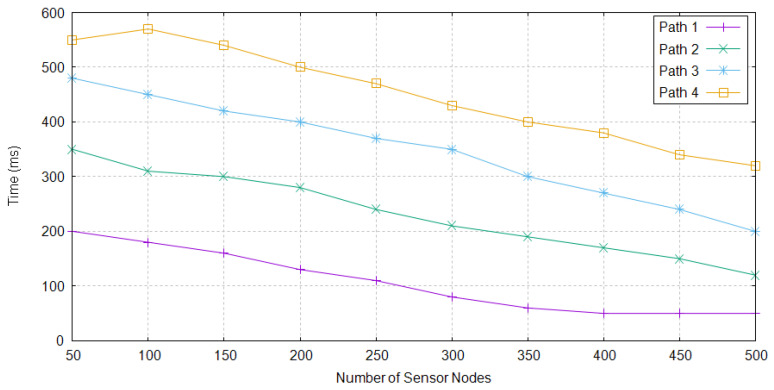
Reliable Paths Creation Time in TBRL.

**Figure 8:**
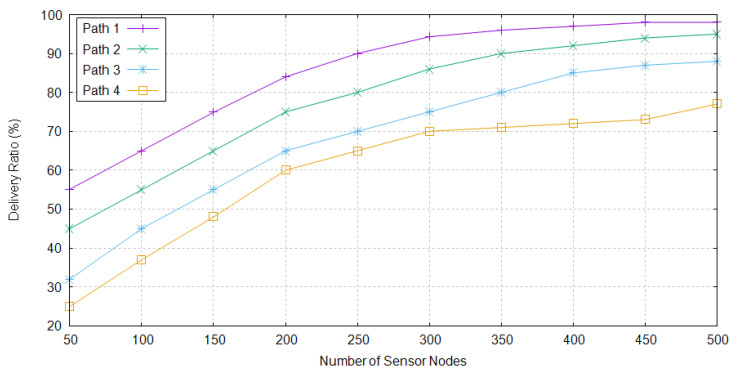
Packet Delivery Ratio of Different Paths in TBRL.

**Figure 9:**
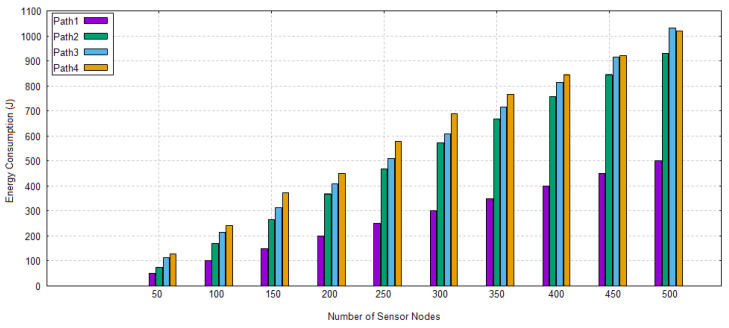
Energy Consumption of Different Paths in TBRL.

**Figure 10:**
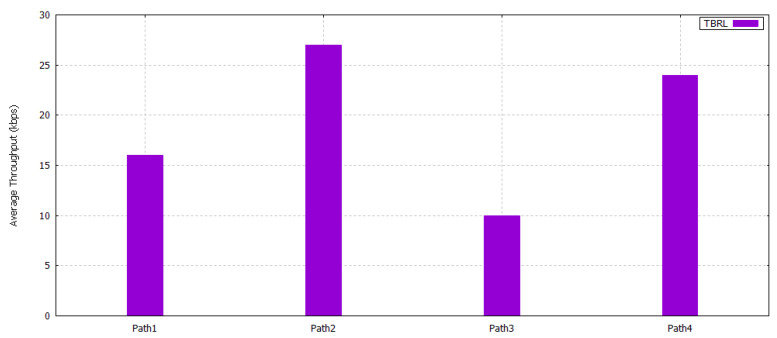
Throughput among Different Paths in TBRL.

**Figure 11:**
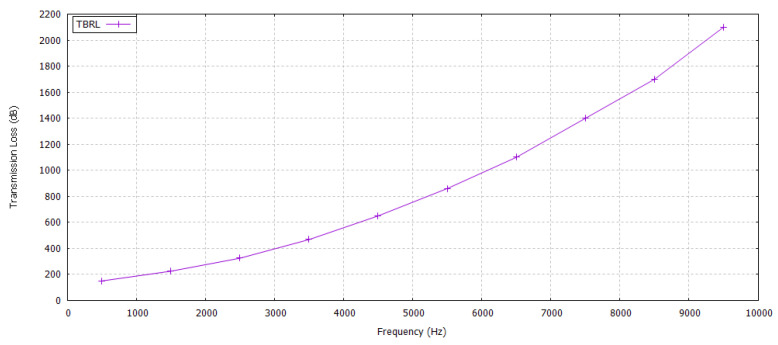
Maximum Transmission Loss in TBRL.

**Figure 12:**
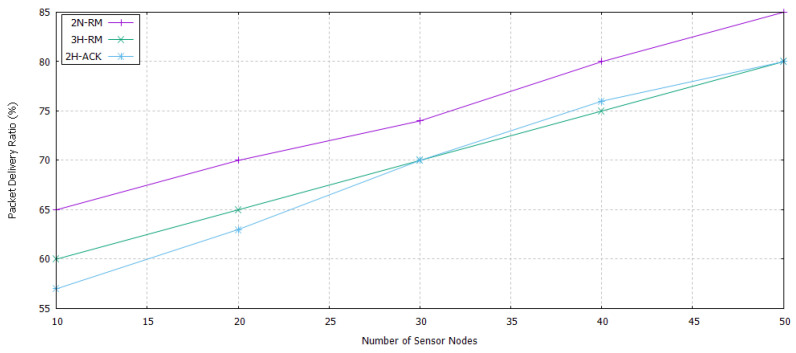
Packet Delivery Ratio of 2N-RM, 3H-RM, and 2H-ACK.

**Figure 13:**
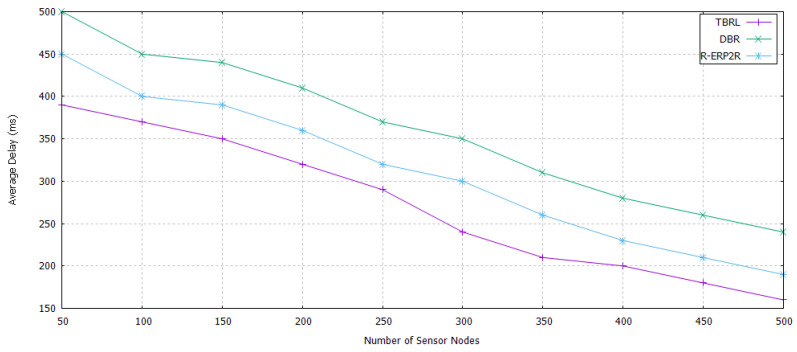
End to End Delay of TBRL, Depth Based Routing (DBR), and Reliable Energy-efficient Routing Protocol (R-ER$P^{2}$R).

**Figure 14:**
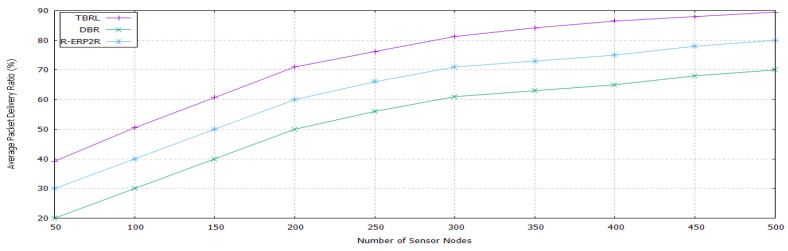
Packet Delivery Ratio of TBRL, DBR and R-ER$P^{2}$R.

**Figure 15:**
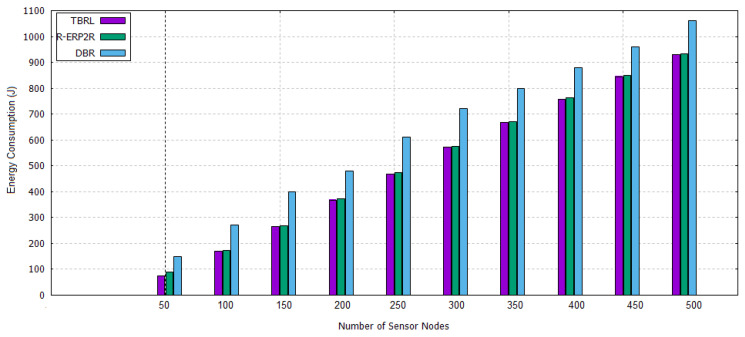
Energy Consumption of TBRL, DBR and R-ER$P^{2}$R.

The simulation results are shown in Figure 7, Figure 8, Figure 9, Figure 10, Figure 11, Figure 12, Figure 13, Figure 14 and Figure 15. The simulation tool is NS-2 AquaSim, which is the extension of NS-2 simulator and specially designed for aquatic environment. Table 4 provides the simulation parameters.The simulation results are explained in two phases: in the first phase, all of the reliable links are established between the nodes at different depth level and these links are evaluated under time, Packet Delivery Ratio (PDR), Energy Consumption (EC), and throughput except. Furthermore, its reliability model is compared in terms of PDR against 3H-RM and 2H-ACK. In the second phase, TBRL compares its performance against DBR and R-ER$P^{2}$R with respect to end to end delay, PDR, and EC of the network.

#### 5.2.1. Time

Time is required in order to make a path among different nodes dataset to reach at sink nodes. For the small set of nodes, maximum time is required to create a path from source to the destination node. As the number of nodes increases, the selection of next node becomes easy, therefore less time took by TBRL. For the selection of path from multiple path environment, TBRL only considers the path having less time required to forward the data packets. Figure 7 shows different paths with respect to the time taken.

#### 5.2.2. Packet Delivery Ratio

Packet delivery ratio shows a successful packet delivery ratio. It is defined as the total number of packets sent from all source nodes over total number of packets received at all sink nodes. When maximum nodes are present in the network, as shown in Figure 8, delivery ratio of TBRL approximately reaches to 99%, whereas, at small number of nodes, data delivery ratio is not good. Distance plays a major role for these results, fewer nodes means that there is higher distance between them, therefore the probability of packets dropped becomes high.

#### 5.2.3. Energy Consumption

The nodes consume energy throughout their lifetime, called energy consumption of nodes. The sum of energies consists of their packet sending, packet receiving, sleeping, and idle state power consumption. These values are initialized through energy model. Furthermore, the mathematical model of energy consumption has been explained in the previous section. Equation (47) is used to determine the value of energy consumption.

(47) TE=TD∗DPE+2.4∗HPS+SNR

TE, TD, DPE, HPS, and SNR represent the total energy consumption, total data packets, data packet energy, hello packet sending, and *SNR*. A network is designed against different set of nodes and their energy consumption is analyzed through Section 4.1. Nodes make a path that has a less amount of energy consumption alive and longer in the network; therefore, path1 is chosen. Figure 9 depicts the energy consumption of paths.

#### 5.2.4. Throughput

Throughput is defined as successful packets delivery at the sink node. It only considers the packets receiving without knowing the total number of packets sent. In TBRL, different paths have different throughput values. Path 2 and 4 have higher throughput as compared to paths 1 and 2, as shown in Figure 10. TBRL is sensitive against delay and packet delivery ratio; therefore, it chooses the throughput value of path 1 because of its less delay and highest packet delivery ratio explained in previous sections.

#### 5.2.5. Transmission Loss

Transmission loss is measured using Equation (48).

(48) $$TL=20logd+\alpha *d*10^{-3}$$

where TL is measured in dB, the distance between sender and receiver *d* is expressed in meters, α, and the absorption coefficient is measured in dB/km. Equation (48) specifies that the transmission loss is mainly caused by distance dependent attenuation and frequency dependent absorption. Transmission loss badly impacts the receiver’s performance. It increases with an increase in attenuation loss and the transmission distance. An acoustic system may operate in a frequency range between 10 and 15 kHz. Although the total communication bandwidth is very low (5 kHz), the system is in fact wideband, in the sense that bandwidth is not negligible with respect to the center frequency. In TBRL, the possible maximum distance exists between two reliable nodes is 70 m; therefore, we took *d* as a 70 m and has transmission loss against different frequencies, as shown in Figure 11.

### 5.3. Packet Delivery Ratio of 2n-Rm

In 3H-RM, there is no mechanism introduced for the acknowledgment process when step size is less than 3 and data packet has been reached at the sink node. Similarly, in 2H-ACK, when the second node becomes sink, it has no choice for taking acknowledgment from other receivers and send it to the source node. Source node waits 60 s for the acknowledgment, after that it will flood the data packet again towards the next node. Consequently, a cyclic process starts between sink and source node that will increase the energy consumption, duplication of data at the sink node, and end to end delay of the network. 2N-RM consists of two nodes reliability models. Its hello packet and acknowledgment packet format is lighter than these two models. When data reached at sink node, there is a zero probability of duplication data packet at sink node due to one to one relationship.

#### Packet Delivery Ratio

The PDR of all these models is presented in Figure 12. PDR is increased as the number of nodes increases. 3H-RM and 2H-ACK have almost same PDR due to their same procedure and participation of more than two nodes for the reliability measurement. Whereas in 2N-RM only two nodes ensure the reliability by taking the consideration of ETX value. Consequently, 2N-RM has higher PDR as compared to 3H-RM and 2H-ACK.

### 5.4. Performance Analysis of Tbrl with Dbr and R-ER$P^{2}$R

For taking the comparison of our proposed routing protocol, we have taken DBR protocol and R-ER$P^{2}$R. DBR uses a greedy approach against nodes depth and finds the next node. A node having less depth as compared to other nodes has been selected as next forwarder. Its greedy nature makes it a favorable choice for managing dynamic topology. R-ER$P^{2}$R uses greedy approach to select the next one, but it also considers the physical distance and residual energy value of next node. It also ensures the quality of a link. Therefore, we have evaluated our results against DBR and R-ER$P^{2}$R. The same datasets of nodes and simulation settings are placed against them and collected results in the form of delivery ratio, time, and energy consumption.

#### 5.4.1. End to End Delay

A comparison against the time needed to create a path from source to the destination node for sending data packets is shown in Figure 13. In some simulation results, DBR consumes less time when compared to TBRL, whereas, in remaining results, it consumes high time. This is caused by the greedy approach that is adopted by DBR. Sometimes favorable results are obtained through DBR. After taking the average of favorable and not favorable results, values have been placed against TBRL. In R-ER$P^{2}$R, a maximum range of physical distance is not set, it takes greedy approach to choose the next one and only makes one path for sending the data. In TBRL, physical distance is set and it takes into account multiple possible paths from source to sink, after that it sorts the path and chooses the optimal one in terms of delay.

#### 5.4.2. Packet Delivery Ratio

The delivery ratio of TBRL against DBR and R-ER$P^{2}$R is shown in Figure 14. Both protocols hold the data packets till found the best suited next node whereas in TBRL, reliable nodes and paths are already known and sorted through IDFS. Therefore, delivery ratio of TBRL is high.

#### 5.4.3. Energy Consumption

Each time, DBR and R-ER$P^{2}$R calculate nodes depth, physical distance, residual energy, and link quality of receiver nodes for the selection of best one among them. Multiple receivers are entertained that cause unnecessary energy utilization in the form of packet receiving, change nodes state from sleeping to alive, and idle to alive power. Whereas, in TBRL, multiple receivers could not be entertained; therefore, its energy consumption is less as compared to DBR and R-ER$P^{2}$R as shown in Figure 15.

## 6. Conclusions

In I-UWSN and smart environment, establishing reliable links is a major requirement. Therefore, we have investigated the parameters that are affected due to poor reliability of links. The significant factor of the poor link reliability is dynamic topology and the effected parameters are transmission loss, packet delivery ratio, network lifetime, and throughput. Dynamic topology occurs due to the continuous flow of water. Therefore, establishing a reliable link for a long time is not possible. Short time reliability can be achieved, therefore, in this article, we have proposed TBRL for dynamic topology routing protocol. Initially, TBRL discovers the approximate location of each sensor node using a topology discovery algorithm. To determine nodes location, only nodes depth is considered as an input parameter. After that, TBRL determines the link reliability of each next possible forwarder by taking their distance, ETX and residual energy level. TBRL continues to establish a reliable link until the sink is reached. In this way, multiple reliable paths from source to sinks are established. On the basis of maximum number of nodes participation, the most reliable path has been selected. For assigning the time interval, average time calculation is considered and that average time is assigned to two nodes reliability model to select the next forwarder. We have evaluated the performance of our proposed protocols with DBR and R-ER$P^{2}$R in terms of packet delivery ratio, end to end delay, throughput, and energy consumption of network. We also tested 2N-RM against 2H-ACK and 3H-RM in terms of packet delivery ratio. The simulation results show that TBRL performs better as compared to the state of the art techniques. It is observed from the results that TBRL is a better protocol for underwater sensor communication and sensor monitoring to support smart cities’ vision. In the future, we will use machine learning techniques for more optimized path selection.

## Figures and Tables

**Table 1:** Comparison with state of the art routing protocols.

Protocol Name	Weakness	Proposed TBRL
GEDAR [22]	The selection of neighboring sets has some trade-offs due to its greedy approach mechanism	It does not support greedy approach, it accepts nodes by calculating reliability in term of expected transmission count, residual energy and distance in between them
DCR [23]	For every time the depth is calculated for flooding, so large end-to-end delays occurred	At initial stage, TBRL determines the depth of each node and create possible paths to avoid large end to end delay
SWARM-SEA [24]	The scheme runs in hybrid mode (DCR + GEDAR), and complexities are increased to detect in underwater nodes movements	It proposed algorithm for detecting the nodes movement to reduce the complexity in term of computational cost
VARP [25]	Longest propagation delay occurs, at every hop count	It proposed average time calculation process in its mathematical model to reduce propagation delay
L2-ABF [26]	Before the flooding, each time angle zone is calculated whereas nodes depth not considered for selection of nodes, so the unexpected delays and energy consumption occur	All reliable links are established and average time calculation is taken, furthermore, specific depth threshold is defined for next node selection. Thus, it helps to measure expected delay and energy consumption.
AHH-VBF [27]	Consumption of battery issues are highly affected	Energy consumption model is provided for the approximate energy utilization of network
NADEEM [28]	Low network throughput, fails to find another routing path when the void nodes are present on a dedicated path	Expected transmission count is considered for the selection of reliable link to maximize network throughput
ASEDG [29]	Link quality and AUVs time for data gathering process not considered, thus unexpected delay occur	Link quality is measured for data gathering process and expected delay is measured
DBR [30]	Used greedy approach in term of depth and not considered links quality between nodes	Next node selection based on link quality and depth threshold is defined for routing process
R-ER*P*^2^R [31]	Maximum range of physical distance is not set, it takes greedy approach to choose the next one and only makes one path for sending the data	Physical distance is set and it takes account multiple possible paths from source to sink, after that it sorts the path and chooses the optimal one
3H-RM [32]	There is no mechanism introduced for the acknowledgment process when step size is less than 3 thus it makes higher probability of packets duplication at sink node	2N-RM consists of two nodes reliability model and no step size is taken. When data reached at sink node, there is a zero probability of duplication data packet at sink node due to one to one relationship.
2H-ACK [33]	When second node becomes sink, it has no choice for taking acknowledgment from other receiver and send it to source node	Two nodes ensure the reliability by taking the consideration of ETX value and have proper mechanism to acknowledge the source node.

**Table 2:** Parameters for Energy Consumption.

HPS	Hello Packet Sending
HPR	Hello Packet Receiving
HPRA	Hello Packet Receiver Acknowledgment
DPE	Data Packet Energy Consumption
N	Number of Iterations
TE	Total Energy Consumed
$E_{i}$	Energy Consumed at ith Iteration
TD	Total Data Packet

**Table 3:** Physical layer model.

Parameter	Value
NL	70 dB
DI	3 dB
SNR	20 dB

**Table 4:** Simulation Setting.

Parameter	Value
Area	1000 × 1000 m${}^{2}$
Nodes Data Set	50, 100, 150, …, 500
Sink Nodes	5
Data Packet Size	1000 Bytes
Communication Medium	Wireless
Wireless Channel	Radio and Acoustic
Radio Speed	3 × $10^{8}$ m/s
Acoustic Speed	1500 m/s
Transmission Range	70 m
Frequency	15 KHz
Energy	Energy Model
Initial Energy	1000 J
Transmission Power	0.5 W
Receiving Power	0.1 W
Idle Power	0.008 W
Sleeping Power	0.01
Physical Layer	UnderwaterPhy
Mac Layer	UnderwaterMac
Antenna	OmniAntenna
Nodes Mobility	Random
Network Topology	2D
